# Characterization of blunt chest trauma in a long-term porcine model of severe multiple trauma

**DOI:** 10.1038/srep39659

**Published:** 2016-12-21

**Authors:** K. Horst, T. P. Simon, R. Pfeifer, M. Teuben, K. Almahmoud, Q. Zhi, S. Aguiar Santos, C. Castelar Wembers, S. Leonhardt, N. Heussen, P. Störmann, B. Auner, B. Relja, I. Marzi, A. T. Haug, M. van Griensven, M. Kalbitz, M. Huber-Lang, R. Tolba, L. K. Reiss, S. Uhlig, G. Marx, H. C. Pape, F. Hildebrand

**Affiliations:** 1 Department of Orthopaedic Trauma, RWTH Aachen University, Germany; 2 Harald Tscherne Research Laboratory, RWTH Aachen University, Germany; 3 Department of Intensive Care and Intermediate Care, RWTH Aachen University, Germany; 4 Chair for Medical Information Technology, Helmholtz-Institute for Biomedical Engineering, RWTH Aachen University, Aachen, Germany; 5 Department of Medical Statistics, RWTH Aachen University, Germany; 6 Medical School, Sigmund Freud Private University, Vienna, Austria; 7 Department of Trauma-, Hand- and Reconstructive Surgery, University of Frankfurt/Main, Germany; 8 Experimental Trauma Surgery, Department of Trauma Surgery, Klinikum rechts der Isar, Technical University of Munich, Germany; 9 Department of Orthopedic Trauma, Hand-, Plastic-, and Reconstructive Surgery, University of Ulm, Germany; 10 Institute for Laboratory Animal Science and Experimental Surgery, RWTH Aachen University, Germany; 11 Institute of Pharmacology and Toxicology, RWTH Aachen University, Germany

## Abstract

Chest trauma has a significant relevance on outcome after severe trauma. Clinically, impaired lung function typically occurs within 72 hours after trauma. However, the underlying pathophysiological mechanisms are still not fully elucidated. Therefore, we aimed to establish an experimental long-term model to investigate physiological, morphologic and inflammatory changes, after severe trauma. Male pigs (*sus scrofa*) sustained severe trauma (including unilateral chest trauma, femur fracture, liver laceration and hemorrhagic shock). Additionally, non-injured animals served as sham controls. Chest trauma resulted in severe lung damage on both CT and histological analyses. Furthermore, severe inflammation with a systemic increase of IL-6 (p = 0.0305) and a local increase of IL-8 in BAL (p = 0.0009) was observed. The pO_2_/FiO_2_ ratio in trauma animals decreased over the observation period (p < 0.0001) but not in the sham group (p = 0.2967). Electrical Impedance Tomography (EIT) revealed differences between the traumatized and healthy lung (p < 0.0001). In conclusion, a clinically relevant, long-term model of blunt chest trauma with concomitant injuries has been developed. This reproducible model allows to examine local and systemic consequences of trauma and is valid for investigation of potential diagnostic or therapeutic options. In this context, EIT might represent a radiation-free method for bedside diagnostics.

The majority of severe chest traumas are associated with significant concomitant injuries such as long bone fractures, abdominal injuries and hemorrhagic shock^1^,^2^. Under these conditions, the posttraumatic course and outcome are significantly influenced by thoracic injuries, which can account for up to 25% of trauma-related deaths^3^,^4^. Therefore, chest trauma represents a leading cause of adverse outcome after multiple trauma^5^,^6^,^7^,^8^. In particular lung contusions are independently associated with posttraumatic complications, such as acute respiratory distress syndrome (ARDS) or multiple organ dysfunction syndrome (MODS)^8^,^9^,^10^,^11^,^12^. Besides the direct impact of chest trauma on pulmonary function, the lung is also an indirect target organ for secondary damage that is caused by the inflammatory response after trauma^13^. Thus, tissue damage may be induced by the traumatic insult itself, as well as by ‘secondary hits’ including therapeutic interventions (e.g. mechanical ventilation) that are frequently required for the treatment of trauma-related respiratory insufficiency^8^,^12^,^14^,^15^.

An increased shunt circulation due to alveolar hemorrhage and atelectasis, with an associated decrease of ventilated areas, is an important cause of early hypoxia after severe chest trauma^11^,^16^. Because of this central role of pulmonary shunting, electrical impedance tomography (EIT) might be a promising diagnostic tool to provide real-time data on lung ventilation, dynamic changes of regional ventilation, and (possibly) future changes in ventilation and perfusion. Further advantages of this technique are its non-invasiveness and the absence of ionizing radiation^17^,^18^,^19^.

Although the clinical relevance of thoracic injuries in multiple trauma patients is apparent, particularly the long-term progression of ventilation changes, as well as the physiological, morphologic and inflammatory changes after blunt chest trauma are not yet fully understood. Despite results from basic research that revealed interesting findings in the very early posttraumatic phase^11^,^20^, experimental settings are often limited by a relatively short observation time^12^. However, it is well known that multiple trauma patients regularly show a clinically relevant aggravation of pulmonary function within the first 3 days after trauma^21^,^22^,^23^. Therefore, this study aimed to develop a long-term multiple trauma model with an observation period of 3 days to characterize the physiological, morphological and inflammatory changes at a later phase after blunt chest trauma. As the porcine physiologic response to severe trauma simulates the human post-traumatic situation more closely than any other non-primates, the experiments were performed in pigs.

## Results

### Hemodynamics and Physiological Response

An average amount of 43% (±6%) of total blood volume was withdrawn for induction of shock. Signs of severe shock were observed in all 12 animals of the PT group (Table 1). A pneumothorax was observed in two animals, one of which developed a tension pneumothorax; these animals were immediately treated by means of a chest tube. All 12 animals survived the 72-h observation period.

**Figure 1:**
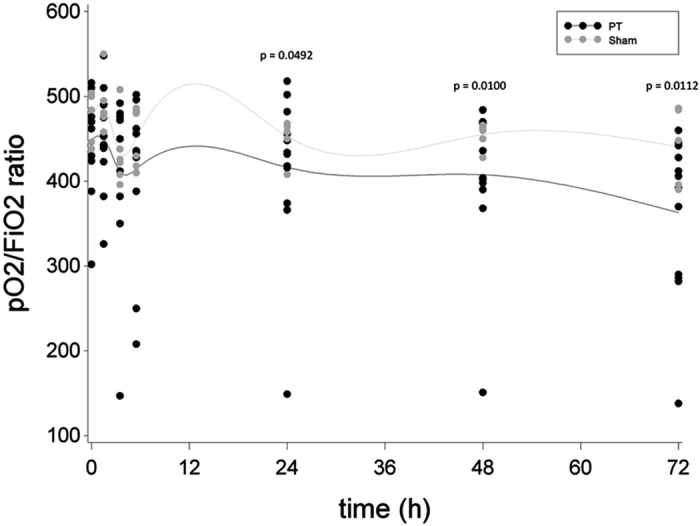
Progressive decrease in the pO_2_/FiO_2_ ratio in the Polytrauma (PT) but not in the Sham group approximated by a cubic spline function with mean values as nodes. Black dots indicate individual values of PT animals at each measurement moment, grey dots indicate individual values of sham animals at each measurement moment.

Progressive lung impairment in the PT group (p < 0.0001, t-value = −5.07, DF = 77) but not in the Sham group (p = 0.2967, t-value = −1.05, DF = 77) was demonstrated by a continuous decrease in the mean pO_2_/FiO_2_ ratio during the observation period (Fig. 1). Compared to sham animals, significantly lower levels of the pO_2_/FiO_2_ ratio were found in the PT group at 24 h (mean pO_2_/FiO_2_-ratio of 416 mmHg (±96) in PT vs. 453 mmHg (±23) in sham animals, 95% CI [−34.98; −0.06], p = 0.0492, t-value = −2.00, DF = 77) as well as 48 h (mean pO_2_/FiO_2_-ratio of 408 mmHg (±89) in PT vs. 455 mmHg (±14) in sham animals, 95% CI [−60.80; −8.54], p = 0.0100, t-value = −2.64, DF = 77) and 72 h (mean pO_2_/FiO_2_-ratio of 363 mmHg (±96) in PT vs. 441 mmHg (±46) in sham animals, 95% CI [−92.40; −12.23], p = 0.0112, t-value = −2.60, DF = 77) compared to sham animals.

### Computer tomography

**Figure 2:**
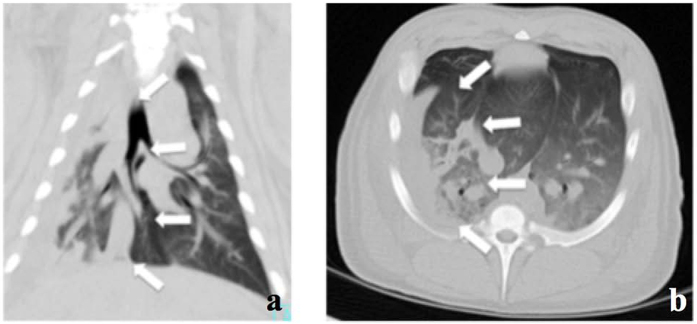
(**a**) CT of right lung (coronary view) with signs of lung contusion (white arrows). (**b**) CT of right lung (horizontal view) with signs of lung contusion (white arrows).

Trauma led to severe signs of lung contusion and reproducible damage of 25–35% of right lung tissue on computer tomography (CT) morphological analysis (Fig. 2a and b). Typically, non-segmental areas of consolidation and ground-glass opacification were observed and rib fractures (2–3 ribs) were found over the traumatized thorax.

### Posttraumatic Inflammation

**Figure 3:**
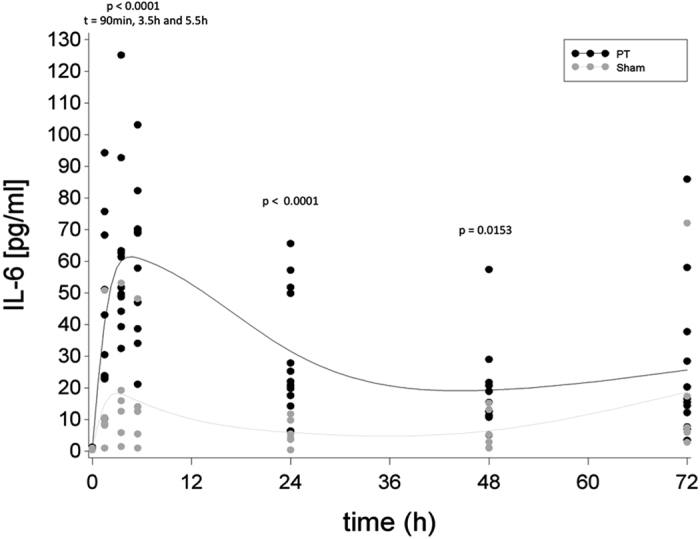
Levels of systemic interleukin (IL)-6 over time ratio in the Polytrauma (PT) and the Sham group approximated by a cubic spline function with mean values as nodes. Black dots indicate individual values of PT animals at each measurement moment, grey dots indicate individual values of sham animals at each measurement moment.

**Figure 4:**
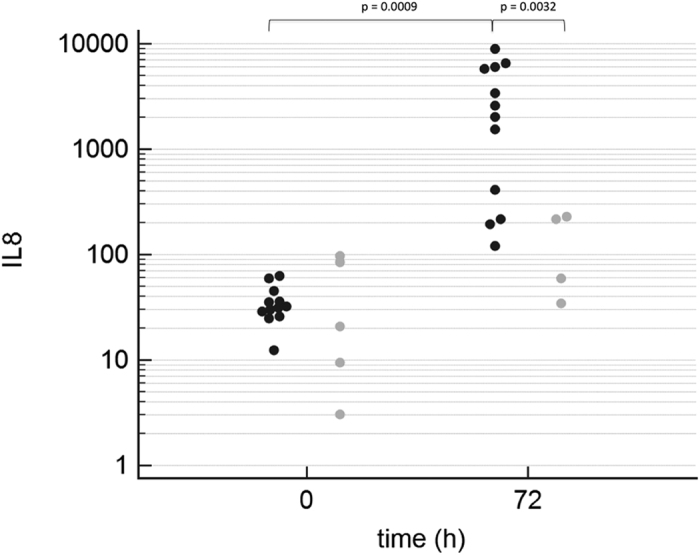
IL-8 Concentrations of IL-8 in bronchoalveolar lavage (BAL) fluid in logarithmic scale before (0 h) and 72 h after induction of trauma. Black dots indicate individual values of PT animals at each measurement moment, grey dots indicate individual values of sham animals at each measurement moment.

After a 90-min period of shock, mean systemic IL-6 concentrations in the PT group showed a significant increase over time (p = 0.0305, t-value = −2.20, DF = 90) with the highest IL-6 levels at 5.5 h post-trauma (61 pg/ml (±23), Fig. 3). In contrast, in the Sham group, changes in mean systemic IL-6 concentration showed a non-significant increase over time (p = 0.8008, t-value = 0.25, DF = 90). For mean local IL-8 concentrations in BAL fluid, compared to baseline, the PT group showed a significant increase at 72 h (26 pg/ml (±24) vs. 1 pg/ml (±0), p = 0.0009, t-value = 4.30, DF = 13) and also compared to the Sham group (26 pg/ml (±24 vs. 19 pg/ml (±27), p = 0.0032, t-value = 9.23, DF = 13, Fig. 4).

### Lung Injury

**Figure 5:**
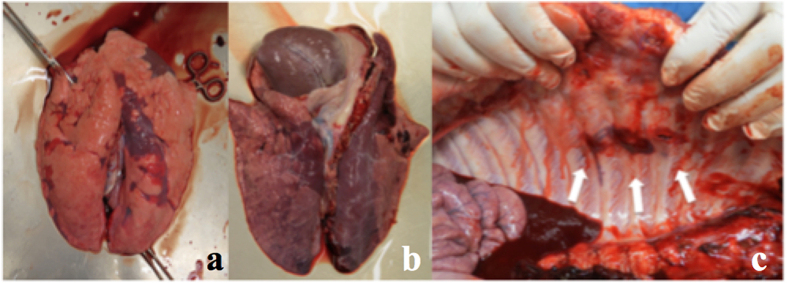
(**a**) Lung from Sham group. (**b**) Traumatized lung. (**c**) Rib fractures (white arrows).

**Figure 6:**
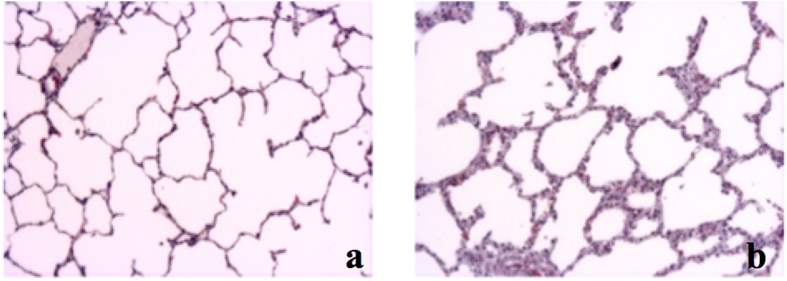
(**a**) Sham animal. (**b**) Polytrauma animal.

In accordance with radiologic and inflammatory analysis, macroscopic and histologic evaluation also revealed severe signs of lung damage with diffuse alveolar damage (Figs 5a–c and 6b). A significant increase of mean interstitial thickness associated with edema^24^,^25^ was found in injured lungs compared to lungs from the Sham group (Sham 0.5 points (±0.53) vs. PT 1.25 points (±0.79), p < 0.0001, F-value = 16.56, DF = 4/15) (Fig. 6a and b).

### EIT Measurements

**Figure 7:**
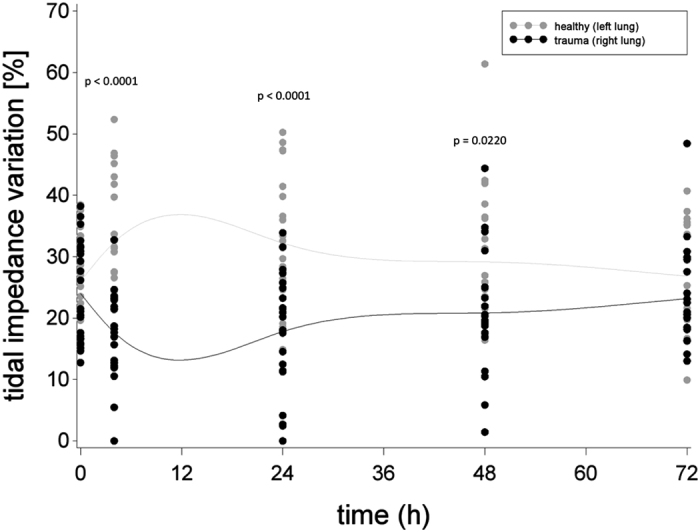
Tidal impedance variation (%) in traumatized (right lung) and non-traumatized (left lung) parenchyma before shock (0 h) and up to 72 h thereafter, approximated by a cubic spline function with mean values as nodes. Black dots indicate individual values of PT animals at each measurement moment, grey dots indicate individual values of sham animals at each measurement moment.

After trauma, EIT measurements revealed significant differences in mean impedance and, thus, in ventilation between the right lung (trauma) and left lung (healthy) during the observation period (p < 0.0001, F-value = 33.91, DF = 1/140) (Fig. 7).

**Figure 8:**
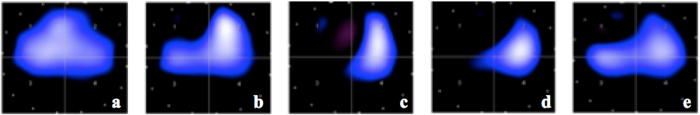
Regions of interest and tidal variation before trauma (**a**), and at 4 h (**b**), 24 h (**c**), 48 h (**d**) and 72 h after trauma (**e**) in a representative animal, see also^82^.

Changes in mean impedance measurements were found between the different ROIs (Table 2; Fig. 8). After induction of chest trauma, lung ventilation was significantly decreased in the traumatized ventral and dorsal areas of the right lung (ROI 1 & 3, Fig. 8b). These differences were still significant after 24 h (Fig. 8c).

## Discussion

According to epidemiologic data, in multiple trauma patients there is a high coincidence of chest trauma, extremity fractures, abdominal injuries and hemorrhagic shock^1^,^2^. Under these conditions, the severity of primary and secondary pulmonary damage is considered an important factor for outcome^26^. However, few studies have focused on long-term pulmonary consequences after multiple trauma. Therefore, we aimed to establish a clinically relevant multiple trauma model that includes long-term ventilation and ICU monitoring. In this model, we focused on characterization of the physiological, morphological and inflammatory impact of blunt chest trauma on lung parenchyma and function in the presence of additional multiple injuries and hemorrhagic shock.

Summary of the main findings:

1. This long-term multiple trauma model with a posttraumatic observation period of 72 h (with mechanical ventilation and ICU monitoring) was shown to be feasible. The clinical relevance is reflected by severe impairment of the physiologic parameters investigated.
2. The model includes a standardized and reproducible chest trauma with 25–35% of contused right lung tissue as determined macroscopically and on CT scanning.
3. The traumatic insult resulted in significant histological pulmonary damage and in a marked pulmonary and systemic inflammatory response.
4. EIT revealed reduced ventilation over the traumatized lung with a compensatory increase of the contralateral uninjured lung.

To the best of our knowledge, this multiple trauma model represents the longest post-traumatic observation period under clinically relevant conditions including mechanical ventilation and ICU monitoring. This long-term model also addressed comments/recommendations arising from previous studies with a shorter observation period (±6–12 h); i.e. these studies emphasized the need for a longer observation period to allow conclusions to be drawn about the characteristics of the later post-injury phase, as well as the effects of long-term ventilation after severe trauma^12^,^27^. Also, in earlier models, either an isolated chest trauma or a combination of chest trauma and controlled hemorrhage was induced^12^. However, such models fail to take into account that most thoracic injuries occur in combination with other relevant injuries. Therefore, we developed a model with several injury mechanisms and/or patterns frequently found in association with blunt chest trauma.

In addition, general recommendations for experimental trauma models to adequately mimic the clinical scenario were made by the 2000 Military Medicine Workshop on Animal Models in Hemorrhage and Resuscitation Research^27^,^28^. These include the need for potential uncontrolled bleeding, surgical procedures coincident with hemorrhage, relevant soft tissue trauma to better approximate the posttraumatic immune response and, finally, a comparable duration of hypotension before resuscitation^27^,^28^. We believe that our model largely fulfills these requirements; particularly because the applied liver laceration can cause uncontrolled bleeding and abdominal packing, and fracture stabilization represents surgical interventions coincident with hemorrhage. In addition, the induction of chest trauma and femoral fracture are associated with significant soft tissue damage; also, the period until resuscitation was taken from the general clinical situation. Moreover, large animal models provide a greater degree of similarity to human trauma scenarios (including cardiovascular, ventilation and inflammatory parameters) and, thus, better applicability of the experimental data to the clinical setting^12^.

Several critical findings from earlier studies are addressed in the present study^8^,^11^,^14^,^29^. On the one hand, in line with other studies, our microscopic findings show significant damage of lung tissue after chest injury^11^,^20^, which is also observed in humans with sustained chest trauma^30^. On the other hand, in contrast to other studies that lacked precise evaluation of the contused pulmonary volume^11^, we performed CT scanning that allows reliable determination of the volume of lung contusion. The CT scans consistently demonstrated severe lesions of 25–35% of pulmonary volume, thereby corroborating the reproducibility and reliability of our model.

Similar to our multiple trauma model, Couret *et al*.^11^ observed an immediate decrease of cardiac output and MAP after isolated chest trauma. Both these studies underline the relevance of thoracic injuries after multiple trauma, as chest trauma-related changes (e.g. hypoxia, edema, altered cardiovascular reflexes) have a significant impact on the post-injury response. In addition to a potential vagal reflex in the very early posttraumatic phase, other mechanisms (e.g. depressed myocardial contractility, myocardial stunning, respiratory acidosis, hypoxic pulmonary vasoconstriction) have been proposed for the depressed hemodynamic situation in the later phase^31^,^32^,^33^,^34^.

In other large animal models, the pO_2_/FiO_2_ ratio has been used to describe pulmonary function. In accordance with other studies^11^,^20^, we also found a significant decrease of the pO_2_/FiO_2_ ratio during the posttraumatic period. However, in some models, paralyzing agents were used to prevent spontaneous breathing^35^, while others used repetitive bolt shots for chest trauma induction^11^,^20^, or did not apply clinically relevant parameters for mechanical ventilation (i.e. decreased FiO_2_ for 5 h, no lung protective ventilation)^36^. These techniques of induction of trauma and the posttraumatic treatment have an impact on lung function and probably decrease the pO_2_/FiO_2_ ratio in a clinically non-relevant way; such measures may also explain why the observation period in most of these studies did not extend beyond 12 h.

Similar to our findings, other long-term studies on the effects of chest trauma also reported no significant changes of the early pO_2_/FiO_2_ ratio in the acute posttraumatic phase, but a progressive impairment of pulmonary function over time^11^,^37^. This dynamic course is comparable to observations in multiple trauma patients that regularly show a clinically relevant aggravation of pulmonary function at day 3 after trauma^21^,^22^,^23^. While an initial decrease of pO_2_/FiO_2_ might be explained by compensatory mechanisms (such as shunting and ventilation/perfusion mismatch during the acute posttraumatic phase) later impairments may be caused by local inflammatory changes inducing a marked increase in lung vascular and epithelial permeability and the translocation of protein-rich edema fluid into the air spaces^16^,^38^,^39^,^40^,^41^. Therefore, we believe that the progressive deterioration with a significant decrease of the pO_2_/FiO_2_ ratio at day 3 after trauma reflects the clinical situation.

Mechanical ventilation has the potential to enhance the primary damage of lung tissue after chest trauma. Both mechanical (e.g. cyclic stretching) and inflammatory aspects have been suggested as possible explanations^42^,^43^. The relevance of mechanical ventilation has been emphasized by studies describing a significant increase of histologic damage in the very early phase (4 h) after blunt chest trauma, whereas no such histopathological changes were observed after chest trauma with spontaneous breathing^29^,^44^. Despite this relevance for secondary lung damage and, although most patients with severe chest trauma require mechanical ventilation, most previous studies either had only a short period of mechanical ventilation or no ventilation at all. In the present study, all animals were ventilated over the entire observational period of 72 h, which (to our knowledge) is one of the longest periods described and close to the mean duration of mechanical ventilation found in large epidemiologic registries of trauma patients^1^.

In the clinical setting, it is well known that blunt chest trauma initiates a relevant local immune response in lung tissue^29^ that is associated with posttraumatic development of ARDS^45^,^46^,^47^. Furthermore, a systemic inflammatory response with increasing cytokine concentrations has been observed and correlated with the incidence of posttraumatic complications (e.g. pneumonia and MODS)^48^. In our model, both local and systemic inflammatory changes were found, supporting its clinical relevance^49^. Nevertheless, despite these similarities to the human situation, the porcine species and the traumatic insult may have had a significant impact on the magnitude and time course of the posttraumatic immune response^12^,^50^.

Relevant hypoxia early after severe chest trauma has often been explained by an increased shunt effect due to alveolar hemorrhage and atelectasis, with an associated decrease of ventilated areas^11^. Accordingly, in a porcine model of lung contusion, Batchinsky *et al*. found improved oxygenation after the pulmonary perfusion was shifted to well-ventilated areas^16^. From these latter results it was concluded that lung contusion induces rapid but transient respiratory impairments due to increased shunt effects^11^. However, early visualization of the respiratory status at the bedside is difficult^51^,^52^. While clinical symptoms like hypoxemia and hypercapnia peak late and are therefore not reliable in the early diagnosis of lung contusion^30^, early visualization of parenchymal changes (e.g. at admission) by plain radiography is known to be unreliable (detection rate of lung contusion 50%)^53^,^54^. This might underestimate the severity of lung contusion and delay appropriate treatment^55^,^56^. Although CT scans represent the most accurate diagnostic tool^57^,^58^ and are the standard diagnostic procedure, they are not always available as a bedside tool during the entire clinical course^59^,^60^,^61^. EIT might overcome these shortcomings by providing continuous evaluation of pulmonary ventilation. In this context, experimental and clinical studies have shown that EIT is a reliable non-radioactive device to determine regional ventilation after non-traumatic insults^62^,^63^. Furthermore, in a rodent model of non-traumatic lung injury, EIT was associated with the severity of pulmonary inflammation^64^. Also, in patients with non-traumatic respiratory dysfunction, a correlation was found between relative impedance changes on EIT images and regional changes of lung air content detected on CT^62^. In that same study, EIT-based adjustments of ventilator settings resulted in enhanced regional ventilation, reduced tidal alveolar collapse and improved gas exchange^62^.

Our results confirm the reliability of EIT also for the difficult setting of chest trauma. In this context, we have shown that EIT visualizes impairment of lung ventilation in contused lung tissue in the very early phase after trauma. Furthermore, EIT revealed inhomogeneous changes of ventilation in different ROIs. Since inhomogeneous ventilation is often seen in patients with lung contusions or ARDS, EIT may help to diagnose trauma patients at risk at a very early state after the insult. Its application during the resuscitation phase respectively the initial operation period and during following intensive care treatment may improve outcome by real-time diagnosis and prompt adaptation of treatment, e.g. by avoiding abnormal distension of primarily unaffected lung sections^65^,^66^. As a growing number of clinical studies implies establishment of EIT under diverse medical conditions^67^, we are certain that this method, once provided in the trauma setting, will help to detect time-displaced lung dysfunction also in trauma patients that suffer from consequences of indirect damage to the lung caused by remote injuries, and multiple organ failure or sepsis. In this context early diagnosis of lung injury and visualization of its dimension (as well adapted mechanical ventilation) are essential for successful treatment, information gained from this new bedside technique may serve to avoid posttraumatic complications^59^.

In the future, technical development of EIT (e.g. three-dimensional imaging) may allow even more detailed imaging of the lung, which will help to diagnose local changes of lung parenchyma after trauma. Furthermore, EIT may help to reduce both the radiation exposure associated with diagnostic procedures and the number of patient transfers^68^,^69^. EIT may even help to differentiate between hydrostatic (i.e. edema) and inflammatory (i.e. fibrin expression) pulmonary changes, thereby aiding therapeutic decisions.

### Limitations

Several limitations of the present model need to be addressed. First, all procedures were performed with animals (PT and Sham) under general anesthesia, which might cause some modulation of cellular injury. Also, although in humans acute trauma does not occur under the conditions described here, ethical guidelines for animals and a moral imperative do not permit any other procedure. In addition, although aiming to provide a clinically realistic setup, the complex injury pattern of our model is influenced by many variables (e.g. drugs, infusions, parenteral diet, ventilation parameters during the clinical course, sample collection, etc.) that may also affect the results. Although the ARDS criteria based on the Berlin definition^70^ were not achieved in our model, a significant decrease of pO_2_/FiO_2_ was observed during the posttraumatic course that is comparable to typical clinical findings with relevant impairment of lung function at day 3 after trauma. Furthermore, macroscopic, histologic and inflammatory analysis clearly revealed the onset of ARDS. Therefore, we suggest that our model adequately reflects the clinical situation. As regional perfusion also plays an important role in the development of posttraumatic shunting, visualization of pulmonary blood flow with a bedside tool comparable to EIT would be desirable. However, this requires further technical standardization and developments.

## Conclusion

We have developed a novel, clinically relevant porcine model of severe multiple trauma (pulmonary contusion, extremity injury, liver laceration) with a relatively long posttraumatic observation period under ICU conditions. This consistent and reproducible model allows to assess diagnostic (e.g. EIT) and therapeutic interventions, as well as their long-term consequences. In this context, EIT may be a promising, non-invasive technique for diagnosing decreased lung ventilation in the acute and late phase after multiple trauma, and for monitoring therapeutic interventions throughout the clinical course.

## Material and Methods

### Animal Care

All experiments were performed in accordance with the German legislation governing animal studies following The *Principles of Laboratory Animal Care*^71^. Official permission was granted from the governmental animal care and use office (Landesamt für Natur, Umwelt und Verbraucherschutz Nordrhein-Westfalen, Recklinghausen, Germany, AZ: 84.02.04.2014A265), which also approved all experimental protocols. Male German landrace pigs from a disease-free barrier breeding facility were housed in ventilated rooms and allowed to acclimatize to their surroundings for a minimum of 7 days before surgery.

All sections of this report adhere to the ARRIVE Guidelines for reporting animal research^72^.

This experiment included 18 male pigs (German Landrace, *Sus scrofa*) 30 ± 5 kg body weight (BW), of which 12 sustained polytrauma (PT group) and 6 served as non-injured controls (Sham group). All animals underwent clinical examination by a veterinarian before the experiments started.

### General Instrumentation and Anesthesia

After a 12-h fasting period with water *ad libidum*, animals were premedicated. For pre-medication animals received an intramuscular injection of 4 mg kg^−1^ azaperone (Stresnil^TM^, Janssen, Germany). Anesthesia was induced by intravenous injection of 3 mg kg^−1^ propofol followed by orotracheal intubation (7.5 ch; Hi-Lo Lanz^TM^). Mechanical ventilation in volume-controlled mode with lung-protective ventilation parameters was applied (6–8 ml/kg/BW); i.e. inspiratory oxygen fraction (FiO_2_) of 0.5; positive end-expiratory pressure (PEEP) 8 mmHg (plateau pressure < 28 mmHg) adjusted by capnometry targeting a pCO_2_ of 35–45 mmHg (Draeger, Evita, Lübeck, Germany) as indicated for the treatment of patients with chest trauma^73^,^74^. Vital parameters were monitored by electrocardiographic (ECG) recordings and ECG-synchronized pulse oximetry, as previously described^75^. Data on vital signs are depicted according to the time points of whole blood sampling.

General anesthesia was maintained with propofol and sufentanil during the entire study period. Fluids were administered by continuous crystalloid infusion (Sterofundin ISO^®^; 2 ml/kg BW/h).

A central venous catheter (Four-Lumen Catheter, 8.5 Fr., Arrow Catheter, Teleflex Medical, Germany) was placed in the external jugular vein for administration of fluids, anesthesia and continuous monitoring of central venous pressure. A three-lumen hemodialysis catheter (12.0 Fr., Arrow Catheter, Teleflex Medical, Germany) was placed in the right femoral vein to induce hemorrhage, and an arterial line (Vygon, Aachen, Germany) was placed in the femoral artery for continuous monitoring of blood pressure, e.g. mean arterial pressure (MAP). All intravascular pressure measurements were referenced to mid-chest level and values were obtained at end expiration. Finally, all animals received a suprapubic catheter (12.0 Fr, Cystofix^®^, Braun, Melsungen, Germany) and were then randomly allocated to either the PT group or the Sham group.

In sham animals, although instrumentation, anesthesia and intensive care management were the same as in the PT group, the Sham group was not subjected to any injury or hemorrhage.

### Induction of Multiple Trauma and Hemorrhage

**Figure 9:**
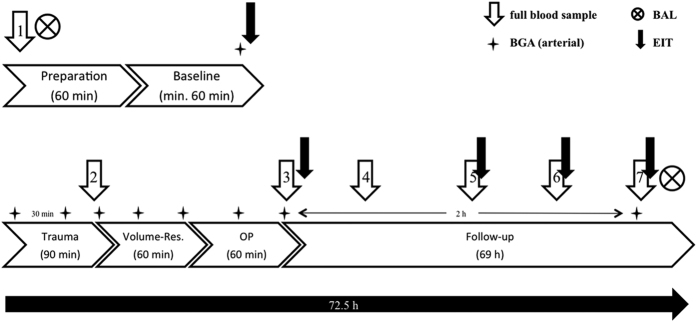
Time line of the study. BAL = bronchoalveoar lavage; EIT = electrical impedance tomography; OP = operative stabilization; BGA = blood gas analysis; Volume-Res. = volume resucitation.

After achieving stable baseline conditions (at least 120 min after instrumentation), animals were subjected to multiple trauma. Before induction of trauma, FiO_2_ was defined at 0.21, simulating ambient air. Furthermore, fluid administration was reduced to 10 ml/h and animals were not prevented from hypothermia for the following 90-min period of shock in order to simulate the clinical situation (in humans) after trauma and transport to hospital (Fig. 9).

Multiple trauma included a femur fracture induced by a bolt gun machine (Blitz-Kerner, turbocut JOBB GmbH, Germany) of which the bolt hit a custom made punch positioned on the mid third of the femur. Cattle-killing cartridges (9 × 17; DynamitNobel AG, Troisdorf, Germany) were used. Furthermore, for blunt chest trauma induction a pair of panels (steel: 0.8 cm and lead: 1.0 cm thickness) was placed on the right dorsal, lower chest. The bolt was shot (Blitz-Kerner, turbocut JOBB GmbH, Germany) onto this panel using cattle-killing cartridges (9 × 17; Dynamit Nobel AG, Troisdorf, Germany) simulating blunt lung contusion as previously described^75^,^76^. The bolt shot was applied while the lungs of the animals were inflated.

Next, a midline-laparotomy was performed and the right upper liver lobe was explored. A penetrating hepatic injury was induced by a crosswise incision (4.5 × 4.5 cm) halfway through the liver tissue^49^,^77^. After a short period of uncontrolled bleeding (30 sec), liver packing was carried out with 5 sterile packs of 10 × 10 cm gauze. After hepatic packing, pressure-controlled and volume-limited hemorrhagic shock was induced by withdrawing blood until a MAP of 40 ± 5 mm Hg was reached, with a maximal withdrawal of 45% of total blood volume. MAP was maintained for 90 min. The “Total Injury Severity Score” was calculated as 27 points. Trauma was induced by one investigator (KH) and the period of shock was monitored by two experienced clinicians (KH; TPS).

At the end of the shock period, animals were resuscitated in accordance with established trauma guidelines (ATLS^®^, AWMF-S3 guideline on Treatment of Patients with Severe and Multiple Injuries^®^) by adjusting FiO_2_ to baseline values, and re-infusing the withdrawn blood and additional fluids (Sterofundin ISO^®^; 2 ml kg/BW/h)^27^. Rewarming was performed using a forced-air warming system until normothermia (38.7–39.8 °C) was reached^27^.

At the end of resuscitation, surgical disinfection and sterile draping was applied to the femur and operative stabilization of the femur fracture was performed according to established trauma guidelines^78^. Reduction and operation of the femur fracture was guided by fluoroscopy (Ziehm Vision, ZiehmImaging, Germany). Antibiotics (Ceftriaxon^®^ 2 g, i.v.) were administered before surgery and then every 24 h until the end of the experiment.

### Electrical impedance tomography

EIT is based on measurement of electrical resistance in various tissues. The electrical conductivity depends on free ion content and differs between biological tissues, and between different functional states of the same tissue. In the lung, bioelectric properties are affected by air content. Consequently, changes in lung ventilation lead to changes in thoracic impedance and to variations in tidal impedance. Inflated lung tissue has a significantly higher resistivity (ρ = 20 Ωm) than after exhalation (ρ = 10 Ωm). Therefore, EIT determines changes in regional intrathoracic impedance that can be correlated with regional pulmonary ventilation changes^79^.

**Figure 10:**
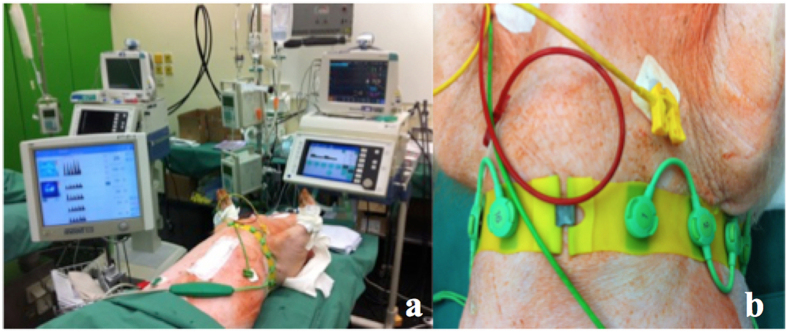
(**a**) shows the electrical impedance tomography (EIT) measurement device. (**b**) placement of the electrodes for EIT.

**Figure 11:**
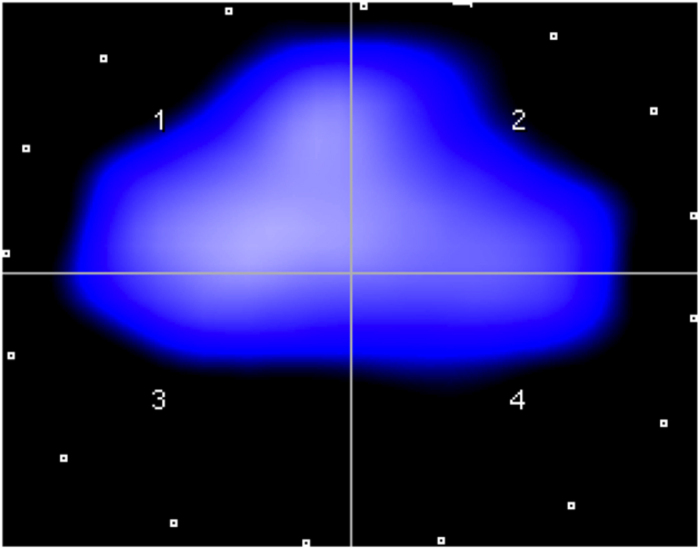
Regions of interest in a typical EIT image. 1: ventral right lung, 2: ventral left lung, 3: dorsal right lung, and 4: dorsal left lung.

To perform EIT measurements, a belt of 16 electrodes (type EIT Evaluation Kit 2 EEK2, Draeger Medical AG & Co KG, Lübeck, Germany) was attached around the animal’s chest (Fig. 10b). An electrical current of low, imperceptible intensity of approximately 5 mA was injected via a pair of adjacent electrodes and the resulting voltages were recorded at all remaining electrode pairs (n = 13). Then, the location of current injection was rotated resulting in 16 × 13 voltage measurements forming the basis for EIT image reconstruction. In the cross-section spanned by the electrode belt, EIT provides information about the magnitude of ventilation in different lung sections (region of interest: ROI), thereby providing the status of regional lung ventilation (Fig. 11). In this way, a two-dimensional image provides information on ventilation in the left and right ventral lung (ROI 1 & 2; Fig. 11) and the left and right dorsal lung (ROI 3 & 4; Fig. 11). EIT measurements were performed using a research EIT device (type EEK2, Dräger Medical AG & Co. KG, Lübeck, Germany) before lung contusion was induced, and at 4, 24, 48 and 72 h after induction of trauma. Variations in tidal impedance were calculated for every ROI based on the determined impedance changes (presented in % ±SD). EIT was analyzed for the traumatic side (right lung) and the unaffected side (left lung).

### Data Collection

For a period of 5.5 h after trauma, pCO_2_, pO_2_, hemoglobin (Hb), base excess (BE), pH and lactate (LAC) were measured every 30 min by blood gas analysis (BGA) (ABL 625; Radiometer, Copenhagen, Denmark). BGA was then performed every 6 h until the end of the observation period. Based on these data, the Horowitz index (pO_2_/FiO_2_ ratio) was calculated. Data on physiologic response (MAP and heart rate; HR) and BGA are presented according to the time points of whole blood sampling.

### Blood processing and interleukin-6 (IL-6) analysis

Whole blood samples were obtained before trauma (0 h), after trauma (1.5 h), after resuscitation and operative treatment (3.5 h), and after 5.5, 24, 48 and 72 h. Samples were kept on ice. Then, after centrifugation at 2000 × g for 15 min at 4 °C, serum samples were stored at −80 °C until analysis of IL-6 concentrations (Quantikine^®^ ELISA kit for porcine IL-6 P6000B; R&D systems, USA) according to the manufacturer’s instructions.

### Bronchoalveoar lavage

Bronchoalveoar lavage (BAL) was performed before induction of trauma (0 h) and at the end of the observation period (72 h) (Fig. 9). Pulmonary concentration of IL-8 in BAL fluid was detected by an ELISA kit (DuoSet ELISA porcine CXCL8/IL-8, DY535, R&D systems, USA).

Finally, after euthanasia, lung tissue samples from both the injured and uninjured lung were collected and fixed by immersion in 4% phosphate-buffered paraformaldehyde. Fixed samples were dehydrated, embedded in paraffin wax, sectioned at 4-μm intervals and stained with hematoxylin and eosin for histologic evaluation. Interstitial thickening was evaluated using a semi-quantitative score (0 = not observed, 1 = mild, 2 = moderate, 3 = marked).

### Sample size calculation

The used number of animals and the allocation ratio of 2:1 is based on logistic and ethical considerations instead of a formal a-priori sample size calculation. With the chosen sample sizes of 12 and 6 in the two groups (PT and Sham) comparable effect sizes as observed in a previous published study on hypothermia in a porcine trauma model^80^ will provide at least 80% power at a significance level of 5%. For example, a two groups Satterthwaite t-test for unequal variances with a 5% significance level based on the reported mean lactate concentration of 3.01 mmol/L (SEM 0.41) in 15 normothermic trauma animals and of 1.02 mmol/L (SEM 0.12) in 5 normothermic sham animals 90 minutes after induced shock will lead to a power of 97% when the sample size in the two groups are 12 and 6 respectively.

### Experimental outcome

All physiological, morphological and inflammatory outcomes characterizing the long-term evolution of severe multiple trauma are equally important to describe the intermodal animal model. Therefore, no distinction between primary and secondary outcome was made.

### Statistical analysis

Analysis of changes within the groups, i.e. Polytrauma (PT) group and Sham group, and comparisons between the groups (PT vs. Sham) over time were performed with a linear mixed effects model^81^ for HR, MAP, LAC, pH, BE, and pO_2_/FiO_2_ ratio as outcomes with random intercept and random slope. The group (PT or Sham) was modeled as fixed effect and an autoregressive covariance structure was applied. For IL-6 as outcome parameter, a similar model with unstructured covariance was fitted to the data. For analysis of the IL-8 data, the model was reduced to the measurement moments at baseline and 72 h. Data on interstitial thickness were analyzed by an unstructured mean model, with measurements at the right and left lung as repeated factor and fixed factor group. In the PT group, EIT measurements were modeled by a linear mixed effects model with random intercept, random slope, and ROI as fixed factor and autoregressive covariance structure. In all models the animal was regarded as the observational unit. Pairwise comparisons between PT and sham animals at specific measurement moments were evaluated by corresponding linear contrasts. Model assumptions and model fit were checked by visual inspection of the residuals, and the measures of influence diagnostics. Observations with strong influence on estimates and their precision were removed from the respective analysis.

Missing values were taken into account by a likelihood based approach within the framework of mixed linear models with the assumptions that missing values are occur at random. For all comparisons the significance level was set at 5%; due to the explorative nature of this study no adjustment was made to the significance level. Results are reported as means and standard deviations (±SD), two-sided p-values were accompanied by values of the test statistic and degrees of freedom (DF). In addition, 95% confidence intervals (CI) for the difference in mean values of particular outcomes between PT and sham animals at specific measurements in time were provided. All analyses were performed with the SAS version 9.4 (PROC MIXED; SAS Institute Inc., NC, USA).

## Additional Information

**How to cite this article**: Horst, K. *et al*. Characterization of blunt chest trauma in a long-term porcine model of severe multiple trauma. *Sci. Rep.* **6**, 39659; doi: 10.1038/srep39659 (2016).

**Publisher's note:** Springer Nature remains neutral with regard to jurisdictional claims in published maps and institutional affiliations.

## Figures and Tables

**Table 1:** Mean values (±SD) of hemodynamic and physiologic parameters for the Polytrauma (PT) and Sham groups of animals.

		0 min	90 min	3.5 h	5.5 h	24 h	48 h	72 h	p-value^a^	p-value^b^
*HR*										
* PT*	***n*** = ***12***	71 ± 12	170 ± 39^c^	86 ± 18^c^	97 ± 22^c^	74 ± 18^c^	79 ± 24	84 ± 23	0.0035^a^	0.0072^b^
* Sham*	***n = 6***	78 ± 14	82 ± 23	85 ± 18	84 ± 18	64 ± 9	64 ± 8	70 ± 16	0.2312^a^	
MAP										
* PT*	***n*** = ***12***	67 ± 6	43 ± 6	78 ± 14	74 ± 11	64 ± 13	80 ± 16	77±16	0.0019^a^	0.0009^b^
* Sham*	***n*** = ***6***	67 ± 6	65 ± 6	74 ± 10	68 ± 5	70 ± 9	82 ± 10^a^	81 ± 15	0.0286^a^	
LAC										
* PT*	***n*** = ***12***	1.54 ± 0.71	4.44 ± 1.42^c^	1.38 ± 0.37^c,d^	1.33 ± 1.64^c^	0.67 ± 0.19^c^	0.73 ± 0.19	0.90 ± 0.56	<0.0001^a^	<0.0001^b^
* Sham*	***n*** = ***6***	1.68 ± 1.14	1.63 ± 0.99	0.97 ± 0.21	0.72 ± 0.13	0.63 ± 0.15	0.60 ± 0.06	0.75 ± 0.19	0.1635^a^	
pH										
* PT*	***n*** = ***12***	7.46 ± 0.03	7.42 ± 0.03	7.49 ± 0.05^d^	7.47 ± 0.08	7.48 ± 0.04	7.50 ± 0.04	7.50 ± 0.04	0.0020^a^	0.0025^b^
* Sham*	***n*** = ***6***	7.47 ± 0.06	7.48 ± 0.05	7.47 ± 0.03	7.48 ± 0.04	7.51 ± 0.01	7.51 ± 0.01	7.50 ± 0.04	0.1060^a^	
BE										
* PT*	***n*** = ***12***	3.81 ± 1.88	0.38 ± 2.25	5.75 ± 1.53^d^	4.83 ± 2.84	5.23 ± 2.46	4.97 ± 1.57	4.31 ± 1.76	0.0902^a^	0.2189^b^
* Sham*	***n*** = ***6***	2.83 ± 1.87	2.57 ± 2.94	4.02 ± 1.81	3.90 ± 1.64	4.55 ± 0.69	3.85 ± 0.49	3.43 ± 1.06	0.6938^a^	

^a^p-value for changes over time per group, ^b^p-value for comparison between polytrauma (PT) and sham over time, ^c^p < 0.05 for PT *vs*. sham, ^d^n = 11 due to temporary technical restrictions with the blood gas analysis device; HR (beats/min), MAP (mmHg), LAC/BE (mmol/l).

**Table 2:** Mean tidal variation (in % ±SD) in the various regions of interest.

Time Point	Ventral Lung		Dorsal Lung	
	trauma	healthy	trauma	healthy
	ROI 1	ROI 2	ROI 3	ROI 4
0 h (n = 12)	30.9 ± 4.7	32.6 ± 4.1	17.0 ± 2.6	19.4 ± 2.9
**p-value, (F-value, DF)**	**0.2738 (1.21, 1/140)**		**0.2241 (1.49, 1/140)**	
4 h (n = 11)	16.5 ± 7.9	37.4 ± 9.9	18.8 ± 6.6	27.2 ± 7.4
**p-value, (F-value, DF)**	**<0.0001 (37.25, 1/140)**		**0.0070 (7.49, 1/140)**	
24 h (n = 11)	17.9 ± 12.0	37.3 ± 8.6	17.7 ± 6.9	27.1 ± 9.5
**p-value, (F-value, DF)**	**<0.0001 (38.06, 1/140)**		**0.0070 (5.19, 1/140)**	
48 h (n = 11)	22.8 ± 11.7	34.5 ± 11.7	18.9 ± 6.8	23.8 ± 5.9
**p-value, (F-value, DF)**	**0.0240 (5.21, 1/140)**		**0.3222 (0.99, 1/140)**	
72 h (n = 10)	27.5 ± 9.2	33.1 ± 5.7	18.8 ± 4.3	20.5 ± 5.1
**p-value, (F-value, DF)**	**0.1761 (1.85, 1/140)**		**0.7859 (0.07, 1/140)**	
